# Efficacy of manual therapy treatments for people with cervicogenic dizziness and pain: protocol of a randomised controlled trial

**DOI:** 10.1186/1471-2474-13-201

**Published:** 2012-10-18

**Authors:** Susan A Reid, Darren A Rivett, Michael G Katekar, Robin Callister

**Affiliations:** 1 The Faculty of Health, The University of Newcastle, Callaghan, NSW 2308, Australia

## Abstract

**Background:**

Cervicogenic dizziness is a disabling condition characterised by postural unsteadiness that is aggravated by cervical spine movements and associated with a painful and/or stiff neck. Two manual therapy treatments (Mulligan’s Sustained Natural Apophyseal Glides (SNAGs) and Maitland’s passive joint mobilisations) are used by physiotherapists to treat this condition but there is little evidence from randomised controlled trials to support their use. The aim of this study is to conduct a randomised controlled trial to compare these two forms of manual therapy (Mulligan glides and Maitland mobilisations) to each other and to a placebo in reducing symptoms of cervicogenic dizziness in the longer term and to conduct an economic evaluation of the interventions.

**Methods:**

Participants with symptoms of dizziness described as imbalance, together with a painful and/or stiff neck will be recruited via media releases, advertisements and mail-outs to medical practitioners in the Hunter region of NSW, Australia. Potential participants will be screened by a physiotherapist and a neurologist to rule out other causes of their dizziness. Once diagnosed with cervciogenic dizziness, 90 participants will be randomly allocated to one of three groups: Maitland mobilisations plus range-of-motion exercises, Mulligan SNAGs plus self-SNAG exercises or placebo. Participants will receive two to six treatments over six weeks. The trial will have unblinded treatment but blinded outcome assessments. Assessments will occur at baseline, post-treatment, six weeks, 12 weeks, six months and 12 months post treatment. The primary outcome will be intensity of dizziness. Other outcome measures will be frequency of dizziness, disability, intensity of cervical pain, cervical range of motion, balance, head repositioning, adverse effects and treatment satisfaction. Economic outcomes will also be collected.

**Discussion:**

This paper describes the methods for a randomised controlled trial to evaluate the effectiveness of two manual therapy techniques in the treatment of people with cervicogenic dizziness for which there is limited established evidence-based treatment.

**Trial registration:**

ACTRN12611000073909

## Background

Dizziness is a very common condition in the community that often leads to physical problems such as unsteadiness and falls, as well as social, emotional and financial issues [1]. There are many causes of dizziness, one being a dysfunction in the upper cervical spine [1-4]. In this condition, termed cervicogenic dizziness, the non-rotary dizziness is described as imbalance or unsteadiness and is related to movements or positions of the neck. Cervicogenic dizziness is accompanied by a range of symptoms including neck pain, neck stiffness, headache, and less often visual disturbances, nausea, ear fullness, sweating, tinnitus, problems with swallowing, temporomandibular joint pain, upper extremity radiculopathy, general weakness and psychological symptoms such as anxiety and disturbances in concentration and memory [5]. Although a disabling condition, there is no established treatment. There is some evidence for manual therapy treatment of this condition but good quality randomised controlled trials (RCTs) are scarce [3,6]. The existence of cervicogenic dizziness has been a topic of some controversy [7,8] but more recent studies and reports have provided evidence in support of its existence [4,9-16].

It has been suggested by Hulse [17] that one third of people with cervical dizziness have their onset due to trauma such as whiplash, one third have an insidious onset following spinal degeneration, and one third are due to other causes. Whiplash injuries are experienced by 0.1% of the population [18] and the incidence of symptoms of dizziness in whiplash sufferers has been variously reported as 20-58% [19], 40-80% [20] and as high as 80-90% [21-23].

Cervical spondylosis, where the cervical zygapophyseal joints are under abnormal mechanical stress, is a major cause of poor balance and dizziness associated with spinal degeneration [19]. This may occur in people with vertebral collapse, decreased cervical disc height or herniated discs, degenerative lesions (e.g. osteoarthritis), inflammatory diseases (e.g. rheumatoid arthritis), vertebral displacement, muscle spasm, or in those wearing cervical collars [19,24,25]. The cervical zygapophyseal joints are the most densely innervated of all the spinal joints [26] with 50% of all cervical proprioceptors occurring in the joint capsules of C1 to C3 [17]. In a study by Colledge et al. [27] investigating the causes of dizziness in the elderly, the authors attributed dizziness to cervical spondylosis in 65% of cases. In support of this theory, extensive degenerative changes such as osteophytes and discopathy on cervical X-rays have been reported in people with this problem [24,28,29]. It is believed that dizziness can also be caused by dysfunction of the deep muscular proprioceptors in the upper cervical spine leading to abnormal input to the vestibular nuclei [19]. In a study by Treleaven, Jull and Sterling in 2003, people with whiplash-associated dizziness and/or unsteadiness (n=102) were shown to have significantly greater joint position errors and a higher neck pain index than control subjects (n=44), consistent with cervical mechanoreceptor dysfunction being a likely cause of the symptoms [30].

### Manual therapy treatments

In 1991, Brian Mulligan, a New Zealand physiotherapist, introduced a physical therapy treatment for cervicogenic dizziness called Sustained Natural Apophyseal Glides (SNAGs) [31]. Although this treatment is used clinically and is accepted in the Physiotherapy profession, there has been very little research to evaluate its efficacy for cervicogenic dizziness. SNAGs have been shown to be an effective treatment for this problem in the short term (12 weeks) [4], however no longer term follow-up of this treatment has been undertaken. Geoff Maitland described another form of manual therapy called passive joint mobilisations that are commonly used to treat neck pain, headaches and other neck problems [32]. A systematic review of the literature showed there is a lack of quality studies evaluating the treatment of cervicogenic dizziness with manual therapy and no studies evaluating the efficacy of Maitland mobilisations in the treatment of this condition [3].

### Study aims

The aim of this paper is to report the study protocol used to investigate the effectiveness of two manual therapy treatments in reducing the symptoms of cervicogenic dizziness and associated pain over 12 months. This will be investigated by comparing the effects of these treatments to each other and to a placebo intervention. Other aims of the study are: 1) to assess the effects of the interventions on cervical range of motion, head repositioning and balance; 2) assess and compare the cost effectiveness of the interventions; and 3) to report any possible adverse effects and treatment satisfaction.

## Methods/Design

A prospective RCT with unblinded treatment and blinded outcome assessment will be conducted in the School of Health Sciences at the University of Newcastle, Australia. Participants (with cervicogenic dizziness) will be randomly allocated to SNAGs, Maitland passive joint mobilisation or placebo groups. Each participant will receive two to six treatments by an experienced physiotherapist over six weeks at the discretion of the treating therapist who will use their clinical judgement to determine the specific dosage based on the participant’s response. Treatment will cease if the participant perceives the condition is adequately improved or if the improvement plateaus, that is, no further improvement is evident over three successive visits.

### Ethics approval

The study design and procedures were approved by the University of Newcastle Human Research Ethics Committee (Protocol Number: H-2009-0377), and the procedures followed were in accordance with the Helsinki Declaration of 1975, as revised in 2000. Written informed consent will be obtained from all participants prior to enrolment in the study.

### Participants and recruitment

**Figure 1:**
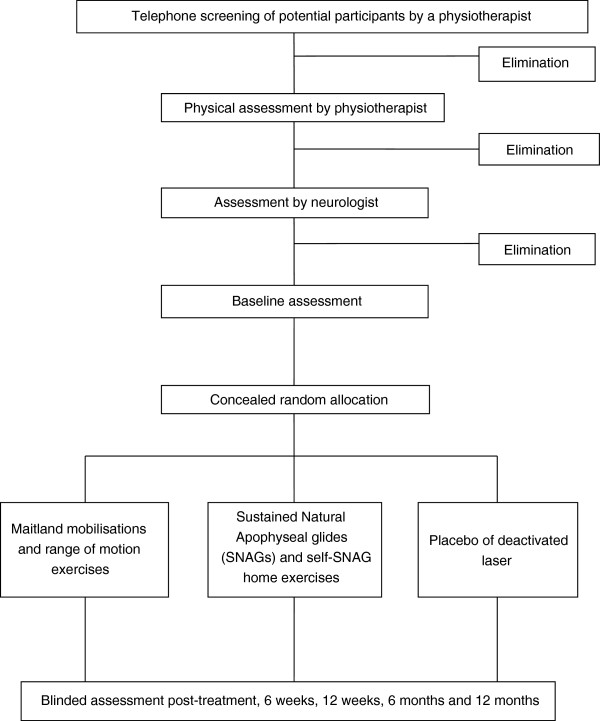
Flow chart of the research protocol.

Ninety participants with cervicogenic dizziness will be recruited in the Hunter region of NSW, Australia, via media releases and resulting radio interviews and newspaper articles, by advertisements in local newspapers, and by referral from medical practitioners including neurologists (Figure 1 Flow chart). Inclusion and exclusion criteria are summarised in Table 1.

**Table 1:** Inclusion and exclusion criteria

**Inclusion criteria:**	
-	has dizziness described as imbalance related to neck movements or positions and a stiff and/or painful neck
-	has had the symptoms present for greater than 3 months
-	18-90 years old
**Exclusion criteria:**	
**Known conditions that would put them at risk of injury:**	
-	inflammatory joint disease
-	spinal cord pathology
-	cervical spine infection
-	bony disease or marked osteoporosis
-	marked cervical spine disc protrusion
-	cervical spine cancer
-	acute nerve root symptoms (severe pain, weakness, pins and needles or numbness in the arm or hand for less than 6 weeks)
-	recent fracture/dislocation of the neck (in the last 3 months)
-	previous surgery to the upper cervical spine
**People will also be excluded if they have the following:**	
-	other types or causes of dizziness, such as vertigo, light headedness, psychogenic dizziness, vertebrobasilar insufficiency
-	other causes of poor balance
-	migraines
-	physiotherapy or similar treatment to the neck in the previous month
-	current pregnancy
-	compensable cases
-	inability to speak or read English

### Screening of potential participants for cervicogenic dizziness

A three step process will be followed to identify people with cervicogenic dizziness. Firstly, an initial phone screening will be conducted by a physiotherapist. Secondly, if potential participants are still thought to have cervicogenic dizziness after the phone discussion they will then be examined physically by the physiotherapist. Thirdly, if not excluded by the physiotherapist at that stage they will have a clinical examination by a neurologist including peripheral vestibular function testing.

#### Phone screening

During the phone screening a history will be taken by the physiotherapist to establish that the person does have dizziness described as imbalance or unsteadiness. If the person has any other types of dizziness such as vertigo, migrainous vertigo, pre-syncope, signs and symptoms of vertebral artery ischaemia (dysarthria, drop attacks, facial paraesthesia, syncope), orthostatic hypotension or psychogenic dizziness [33] they will be excluded. If the dizziness is described as imbalance or unsteadiness it must be established that the imbalance is not due to another cause including musculoskeletal problems, neuromuscular problems, and conditions affecting the brain such as Parkinson’s disease, stroke, cerebellar ataxia, multiple sclerosis, Vitamin B12 deficiency or alcoholism. Then it must be established that there is a related history of neck pain and or/stiffness. The unsteadiness or poor balance must also be exacerbated by cervical spine movements or positions to be considered to be due to cervicogenic dizziness.

#### Physical assessment by a physiotherapist

Those who have passed through the phone screening and are thought to have cervicogenic dizziness then undergo a physical assessment by a physiotherapist. This physical examination includes:

– The Dix-Hallpike manoeuvre performed to determine whether the person has dysfunction of the semi-circular canals [34]. In this test, the participant sits on the examination table while the clinician rotates the participant’s head to 45 degrees then quickly lays the participant straight back so that their head is extended below the horizontal. The production of nystagmus indicates benign paroxysmal positional vertigo.

– Blood pressure measured in sitting and immediately after rising to standing with a digital sphygmomanometer. A normal blood pressure response to positional change will indicate that neurocardiogenic syncope is an unlikely cause of the dizziness [33]. A drop in systolic blood pressure of > 30mm Hg or a drop of 10mm Hg in diastolic blood pressure is indicative of orthostatic hypotension [33].

– Smooth visual pursuit movements assessed by the ability to track a slowly moving object. The examiner looks for asymmetry of eye movement which may indicate a cerebellar lesion [33].

– The vestibulo-ocular reflex with the participant sitting maintaining a gaze on a stationary target and performing small oscillations of the head side-to-side and up-and-down. Abnormal responses such as an inability to maintain the gaze for 60 seconds due to dizziness, blurry vision or double vision may indicate peripheral or central nervous system dysfunction [33].

– Cervical range of motion assessment to determine whether the participant has a restriction of movement which may indicate cervical spine dysfunction consistent with cervicogenic dizziness [5]. The participant is asked to move their neck into flexion, extension, left rotation, right rotation, left lateral flexion and right lateral flexion and report any symptoms such as dizziness or pain.

– Palpation of the upper cervical spine (occiput to C3) performed to identify any stiff and/or painful joints which may indicate dysfunction in the upper cervical spine [5].

– Decreased balance has been reported in people with cervicogenic dizziness [6]. To assess balance, participants will be asked to hold tandem stance for 30 seconds. Tandem stance is a clinical measure of standing balance considered to assess postural steadiness in a heel-to-toe position [34].

#### Examination by a neurologist

If the participant is not excluded after this preliminary screening, they are further assessed by a neurologist to exclude central nervous system, vestibular and other non-cervical causes of the dizziness. This examination will include tests for vestibulo-spinal function, the vestibulo-ocular system, disequilibrium such as gait and balance testing, a repeat of the Dix-Hallpike manoeuvre and peripheral vestibular function testing.

### Randomisation

Participants who were not excluded during the screening process will be randomly allocated to one of three intervention groups: placebo, Mulligan SNAGS and Maitland mobilisations. An independent statistician will produce a computer generated randomisation sequence which will be placed in sequentially numbered opaque sealed envelopes. The randomisation sequence will contain equal numbers of participants in each group.

### Interventions

A physiotherapist with post graduate qualifications and a minimum of 20 years experience in the field of manual therapy will perform all the interventions during the study to all the participants.

#### Placebo

One group of participants will have a placebo intervention consisting of infrared therapy laser which has been deactivated by the manufacturer. A medical laser is commonly used by physiotherapists to treat musculoskeletal symptoms [35]. To the participant, the placebo laser device (a Therapower 40mW laser, serial No 020601, Meyer Medical Electronics, Mordialloc, Australia) will appear to operate normally with a light flashing and a beeping sound, but it will not produce any effective emission. The deactivated laser, which has been shown to have a very strong placebo effect [4,35], will be applied for two minutes to each of three sites on the neck, with the pen at a distance of 0.5-1 cm from the skin [35].

#### Mulligan SNAGs

Another group of participants will receive SNAGs as described by Mulligan [36]. The participant, in the sitting position, is asked to move their head in the direction that particularly produces their symptoms. As the participant moves their head, the physiotherapist gently glides the C1 or C2 vertebra anteriorly and sustains the glide through the movement. During the application of the glide, the participant should stay symptom-free and is instructed to stop moving if any dizziness is produced. This movement is repeated six times at the first treatment session as recommended by Mulligan. At the subsequent treatment sessions provided no dizziness is experienced the SNAG is performed ten times and gentle over pressure can be applied. A second SNAG in another implicated direction of movement may be added to treatment. After the second treatment the participant will be advised to do a self-SNAG (six repetitions) as a home exercise once daily. Written and pictorial instructions for the home exercise will be provided. A second home treatment self-SNAG may be added for another implicated movement direction after the third treatment. The participant will be asked to perform the home exercises once daily for 12 months.

#### Maitland mobilisations

The physiotherapist will palpate the neck to find the three most dysfunctional joints and then perform passive joint mobilisations to those joints (as described by Maitland et al.) [32]. A passive joint mobilisation is where the therapist uses their thumbs to rhythmically apply pressure to a vertebra usually in a posterior to anterior direction. It is usually applied three times for 30 seconds to dysfunctional joints or determined by the clinical judgement of the physiotherapist [32]. After the second treatment the participant will be advised to perform range of motion exercises into flexion, extension, right rotation, left rotation, left lateral flexion and right lateral flexion, three times in each direction, once a day for 12 months. Written and pictorial instructions for the exercises will be provided.

### Outcome measures

Socio-demographic data will be collected at baseline including the participant’s age, gender, and time since commencement of dizziness. The primary and secondary outcomes will be measured at baseline, after the last treatment, at six weeks, 12 weeks, six months (questionnaires only), and one year after treatment is completed. All follow-up assessments will be conducted by researchers blinded to the participants’ group allocation. The researchers conducting the data entry process will be blinded to group allocation.

#### Primary outcome

Severity of dizziness (an average level over the previous week) will be measured with a 100 mm horizontal visual analogue scale (VAS). The VAS has been used successfully to measure dizziness in other studies [4,37-39].

#### Secondary outcomes

1) Frequency of dizziness will be measured on a six-point rating scale (0 = no dizziness, 1 = dizziness less than once per month, 2 = 1–4 episodes of dizziness per month, 3 = 1–4 episodes of dizziness per week, 4 = dizziness once daily, 5 = dizziness more than once a day or constant). This scoring method has been used by several researchers [4,8,37,38] to measure frequency of dizziness.

2) Disability caused by dizziness will be measured with the Dizziness Handicap Inventory (DHI). This is a health status measure specifically designed to assess dizziness. The DHI assesses the quality of life using three subscales evaluating the impact of dizziness on the functional, emotional and physical aspects of everyday life [40]. The highest possible score is 100, indicating maximum self-perceived handicap. The DHI has been shown to be a highly reliable and responsive tool [40-42].

3) Severity of neck pain (an average level over the previous week) will be assessed with a 100 mm VAS. There is much evidence supporting the high validity of the VAS for measuring pain intensity [43-47].

4) Global perceived effect will be used to assess satisfaction with treatment and measured by self-assessment on a six-point scale (0 = no benefit, 1 = minimal benefit, 2 = some benefit, 3 = a lot of benefit, 4 = great benefit, 5 = maximal benefit) as used in other studies [4,48,49].

5) Posturography will be used to identify and quantify disturbances in balance. Body sway will be measured with a Chattecx Balance Dynamic System (Serial No 1001, Chattecx Corporation, the Chattanooga Group, Tennessee). Recordings will be performed during the following tasks.

· standing erect with the neck in the neutral position with eyes open

· standing erect with the neck in the neutral position with eyes closed

· standing erect with the neck extended

· standing erect with the neck in left rotation

· standing erect with the neck in right rotation

· standing on a moving platform.

Posturography has been used in many studies to assess people with dizziness and has been found to have good correlations with the participant’s symptoms [4,6,8,37].

6) A Cervical Range of Motion (CROM) goniometer (Performance Attainment Associates, 3550 Lahore Rd, St Paul, MN), which has been shown to be a reliable tool with good validity [50], will be used to measure cervical spine movements. Active flexion, extension, left and right rotation, and left and right lateral flexion will be measured. Each movement will be measured three times and the average taken.

7) Neck repositioning sense will be assessed with the CROM device. This task tests the participant’s ability to accurately reposition their head and neck. The participant is first seated with their head in a neutral position. They are then asked to close their eyes and to move their head into rotation. At mid-range of rotation, the participant will be asked to stop, hold their head steady and think about their position. This position is referred to as the ‘target position’. After 5 seconds, the participant returns to the starting position and then attempts to find the target position again at which point a reading is taken. The number of degrees difference between the target position and the attempt to find it are calculated. This is performed three times for both right and left rotation and the average taken for each direction of rotation movement.

Information about adverse events will be collected by the treating physiotherapist at each treatment session and by the research assistant at each follow-up measurement session using open-ended questions as per normal clinical practice.

Participants will be given a diary and asked to log medication use, visits to a medical practitioner, visits to other health professionals, time off work, changes in social engagements and adherence to home exercises.

### Data analysis

#### Sample size calculation

The sample size required is based on an analysis using independent t-tests to test for differences between pairs of treatment groups, with alpha set at 0.05. Three comparisons will be made: the SNAG group compared to the placebo group, the Maitland mobilisation group compared to the placebo group and the SNAG group compared to the Maitland mobilisation group. Sample size calculations were based on a difference between the two groups that would be clinically significant for the main outcome measures, supported by the results of previous research where applicable data existed, and clinical expectations for those factors for which no previous data existed. This was estimated by biostatisticians from the Centre for Clinical Epidemiology and Biostatistics, The University of Newcastle, using other studies with the DHI and VAS as outcome measures [41,49,51,52]. The DHI was the primary outcome measure used for sample size calculations, as it is a widely reported measure of self-perceived disability and effect of dizziness on function. It has been shown to have strong validity and short-term test-retest reliability and good internal consistency [53,54]. Visual analogue scales have also been used in many studies to measure pain and the main complaint [49] and been shown to have high reliability and validity, and a calculation of sample size was also based on VAS data.

Assuming that the standard deviation of DHI scores is 15, then 30 participants per group will give the study 80% power to detect a difference of 11 units between groups for each comparison. Thirty participants per group are also required based on a 0–10 VAS scale (e.g. for dizziness), with a standard deviation of 2.4 and a clinically significant difference of 2 units with a power of 80% and a 5% confidence level.

#### Statistical methods

Biostatisticians from the University of Newcastle will guide and assist with the statistical analyses. Baseline characteristics will be summarized per group using the number of observations, mean, standard deviation, median, minimum and maximum for continuous measures and number of observations and frequency for categorical measures. Primary and secondary outcome measures are either continuous or ordinal in nature and will be analysed using generalized linear mixed models. As an example, for the primary outcome measure of the DHI, the outcome variable will be the DHI and the predictors will be time, treatment group and an interaction term for time by treatment group. The p-value for the interaction term will indicate whether there is a statistically significant difference in change in the DHI over time between the groups. We will use the ‘gate keeper’ approach to take account of the multiple testing and restrict the overall type I error rate to 5%. This means that we will test the SNAG intervention against the placebo first, then the Maitland mobilisation against the placebo and if those results are statistically significant at the 5% level we will then test the SNAG against the Maitland intervention. The primary and secondary outcome measures will also be compared between treatment groups at each time point using independent t-tests.

#### Economic evaluation

The type of economic evaluation by a health economist will depend on the results. It is possible that there will be a difference in efficacy, so a cost effectiveness or cost-utility analysis will be appropriate. If one intervention were more effective and less expensive, an incremental cost effectiveness (utility) ratio (ICER) would not need to be calculated as it would be clear that the more effective intervention is preferred. If one intervention were more effective and more expensive, then an ICER would need to be calculated. If the results show that one intervention is equally effective to the alternative(s), then a cost-minimisation analysis is appropriate. In this case, there is no difference in effectiveness so the economic analysis would be a comparison of costs only; if one intervention is cheaper, it is the preferred alternative.

#### Controlling bias

To minimise bias randomisation, concealed allocation, specific inclusion and exclusion criteria, blinded outcome assessment, patient blinding, blind data analysis, and intention to treat analysis have been used. It was not possible to blind the physiotherapist performing the interventions.

## Discussion

This paper outlines the rationale and design for a RCT that compares the effectiveness and cost effectiveness of:

a) SNAGs to a placebo intervention

b) Maitland passive joint mobilisations to a placebo intervention

c) SNAGs to Maitland passive joint mobilisations

in reducing symptoms of cervicogenic dizziness and associated pain over 12 months. The value of this study will be to determine which of two common manual therapy treatments is most effective for this problem and whether manual therapy is effective in the longer term (up to one year). The study will contribute to evidence-based manual therapy leading to improved clinical decision making in this field of clinical practice.

## Competing interests

This study is partly funded by the Mulligan Concept Teachers Association Award. The authors declare that they have no competing interests.

## Authors’ contributions

SR, DR, RC and MK were responsible for the design of the study. All authors read and approved the final manuscript.

## Pre-publication history

The pre-publication history for this paper can be accessed here:

http://www.biomedcentral.com/1471-2474/13/201/prepub
